# Supplementary material for: Improvement of the Polyhydroxyalkanoates Recovery from Mixed Microbial Cultures Using Sodium Hypochlorite Pre-Treatment Coupled with Solvent Extraction
Source: Polymers (Basel). 2022 Sep 21;14(19):3938. doi: 10.3390/polym14193938 (PMC9573287; doi:10.3390/polym14193938)
Supplement: Supplementary file 1 [file polymers-14-03938-s001.zip › polymers-1854717-supplementary.pdf]

polymers

MDPI

Improvement of the polyhydroxyalkanoates recovery from mixed microbial cultures using sodium hypochlorite pre-treatment coupled with solvent extraction

- Supplementary Material -

Item 1. In preliminary experiments, the recovery efficiency of the biopolymers (RE) was optimized using dimethyl carbonate (DMC) at 90°C. The following conditions were evaluated:

- Initial lyophilized biomass concentration (25 – 100 g dry-cell/L)
- Contact time (1 and 3 h) of the biomass with the solvent
- PHA precipitation with ethanol

Known amounts (25, 50 or 100 mg) of the lyophilized biomass were transferred to Eppendorf vials. Then, the samples were resuspended in 1 mL of Milli-Q water and mixed with 2 mL DMC. The vials were incubated for 1 or 3 h at 90 °C. The vials were periodically stirred using a vortex. Then, vials were centrifuged at 6.5× *g* for 15 min at room temperature. Three phases were obtained: 1) a supernatant phase composed by water and water-soluble compounds, 2) an intermediate phase that corresponds to the organic fraction containing the solubilized PHAs, and 3) a precipitate composed by biomass and cell debris. The organic fraction (2) was recovered and filtered through 0.45 μm pore size cellulosic membranes (Millipore®) to remove any cell debris. Finally, the solvent was removed by evaporation to obtain a dried PHA sample.

Precipitation of PHA was performed according to Gahlawat et al. [1] in order to enhance the recovery of PHA from the lyophilized biomass. 2 mL of the filtered organic phase containing PHA was mixed with 6 mL of ice-cold absolute ethanol (96%). Then, the mixture was incubated at room temperature with periodic stirring during 1h. Finally, PHA was obtained by volatilizing the solvents.

Table S.1 shows the effect of the initial lyophilized biomass concentration on the extraction and recovery of PHA using DMC at 90 °C. In the case of batch A (See section 2.1 of the manuscript), when the lyophilized biomass concentration was 25 g/L, the amount of recovered PHB was negligible. A similar result was obtained for samples corresponding to PHBV enriched biomass samples. Table S.1 shows that in the case of PHB, the increase of the lyophilized biomass concentration had a negligible effect on the recovery efficiency (RE). Conversely, RE corresponding to the copolymer reached 17 ± 1 % when 50 g dry-cell/L was used. However, when 100 g dry-cell/L was used, the suspension became very viscous, leading to a quite low RE of the PHAs. Table S.1 shows that the increase in the extraction time from 1 to 3 h did not improve the RE of the polymers. For this reason, a lyophilized biomass concentration of 50 g dry-cell/L, and 1h of extraction time were employed in all the subsequent experiments of the manuscript.

Increasing the recovery efficiencies of PHA from the biomass is a crucial factor to develop an economically viable PHA production process. For this reason, a simple test modification consisted in the precipitation of the PHA using cold ethanol [1]. However, this procedure did not improve the PHA recovery from the lyophilized biomass obtained in batch A or B (Table S.1).

Table S1. PHA recovery efficiencies (RE) using DMC at 90 °C. Depicted values correspond to the average ± one standard deviation of three measurements.

Sample	Biomass concentration (g dry-cell/L)	Precipitation with ethanol	Extraction time (h)	RE (%)
Biomass enriched in PHB	25	no	1	≈ 0
	50	no	1	4 ± 3
	100	no	1	2 ± 1
	50	no	3	5 ± 2
	50	yes	1	8 ± 2
Biomass enriched in P(HB-co-HV)	25	no	1	≈ 0
	50	no	1	17 ± 1
	100	no	1	5 ± 1
	50	no	3	12 ± 3
	50	yes	1	7 ± 5

References

1. Gahlawat, G.; Soni, S.K. Valorization of Waste Glycerol for the Production of Poly (3-Hydroxybutyrate) and Poly (3-Hydroxybutyrate-Co-3-Hydroxyvalerate) Copolymer by Cupriavidus Necator and Extraction in a Sustainable Manner. *Bioresour Technol* **2017**, 243, 492–501, doi:10.1016/j.biortech.2017.06.139.
